# Subtotal cholecystectomy for difficult acute cholecystitis: how to finalize safely by laparoscopy—a systematic review

**DOI:** 10.1186/s13017-021-00392-x

**Published:** 2021-09-08

**Authors:** Adriana Toro, Michele Teodoro, Mansoor Khan, Elena Schembari, Salomone Di Saverio, Fausto Catena, Isidoro Di Carlo

**Affiliations:** 1 General Surgery, Augusta Hospital, Siracusa, Italy; 2 Department of Emergency, S. Marco Hospital, Catania, Italy; 3 Brighton and Sussex University Hospitals NHS Trust, Brighton, UK; 4 Department of General Surgery, Whipps Cross University Hospital-Barts Health NHS Trust, London, UK; 5 General Surgery, University of Insubria, Varese, Italy; 6 Emergency and Trauma Surgery, Parma Maggiore Hospital, Parma, Italy; 7 Department of Surgical Sciences and Advanced Technologies “G.F. Ingrassia”, Cannizzaro Hospital, University of Catania, Via Messina 829, 95126 Catania, Italy

**Keywords:** Gallbladder, Difficulty cholecystectomy, Laparoscopic subtotal cholecystectomy, Complications, Clavien–Dindo classification

## Abstract

**Background:**

Aim of this study was to clarify the best laparoscopic subtotal cholecystectomy (LSTC) technique for finalizing a difficult cholecystectomy.

**Patients and methods:**

A review was performed (1987–2021) searching "difficulty cholecystectomy" AND/OR "subtotal cholecystectomy". The LSTC techniques considered were as follows: type A, leaving posterior wall attached to the liver and the remainder of the gallbladder stump open; type B, like type A but with the stump closed; type C, resection of both the anterior and posterior gallbladder walls and the stump closed; type D, like type C but with the stump open. Morbidity (including mortality) was analysed with Dindo–Clavien classification.

**Results:**

Nineteen articles were included. Of the 13,340 patients screened, 678 (8.2%) had cholecystectomy finalized by LSTC: 346 patients (51.0%) had type A LSTC, 134 patients (19.8%) had type B LSTC, 198 patients (29.2%) had type C LSTC, and 198 patients (0%) had type D LSTC. Bile leakage was found in 83 patients (12.2%), and recorded in 58 patients (69.9%) treated by type A. Twenty-three patients (3.4%) developed a subhepatic collection, 19 of whom (82.6%) were treated by type A. Other complications were reported in 72 patients (10.6%). The Dindo–Clavien classification was four for grade I, 27 for grade II, 126 for grade IIIa, 18 for grade IIIb, zero for grade IV and three for grade V.

**Conclusion:**

In the case of LSTC, closure of the gallbladder stump represents the best method to avoid complications. Careful exploration of the gallbladder stump is mandatory, washing the abdominal cavity and leaving drainage.

## Introduction

Laparoscopic cholecystectomy is considered the gold standard for treatment of benign gallbladder diseases [1]. Cholecystectomy using this method can be completed in 90% of elective cholecystectomies and 70% of emergency cholecystectomies [2]. Acute cholecystitis, especially if difficult, can change the above paradigm, resulting in open conversion or change of technique. The conditions that define a difficult cholecystectomy are as follows: necessity of conversion from laparoscopic to open surgery; duration of procedure greater than 180 min; blood loss greater than 300 ml; and urgent need for involvement of a more experienced surgeon [3].

One of the "rescue" procedures to complete the surgery safely (both for the surgeons and patients) is subtotal cholecystectomy (STC). Open [4–6] and laparoscopic [7–9] subtotal cholecystectomy have been reported. For many surgeons, this is considered a bail out technique [10, 11], and the timing of decision making is crucial to avoid catastrophic complications. The capability to perform STC in laparoscopy is increasingly requested during difficult laparoscopic cholecystectomy. Difficult LC has a risk of BDI from 3 to 5 times higher in laparoscopy than open surgery. In case of operative difficulties of young surgeons mostly trained in laparoscopy the help of senior surgeons is strongly recommended [12].

The purpose of the present study is to clarify how laparoscopic subtotal cholecystectomy may be used to complete a difficult cholecystectomy for acute cholecystitis without serious complications.

## Patients and methods

A systematic literature review was performed using the PubMed, Cochrane and Google Scholar databases, in accordance with the PRISMA guidelines [13], to identify published studies from 1987, the date of the first published laparoscopic cholecystectomy, through January 2021**.** The terms used in our research were "difficulty cholecystectomy" AND/OR "subtotal cholecystectomy". All abstracts were read. Systematic reviews, meta-analyses, case reports, letters, articles not written in the English language and articles on animals were excluded. Articles regarding cirrhotic patients, portal hypertension, Mirizzi syndrome, and gallbladder cancer were excluded. Articles in which the type of technique for subtotal cholecystectomy and complications were not carefully described were excluded.

A resident from the Department of Surgical Sciences and Advanced Technologies "G.F. Ingrassia", University of Catania, Cannizzaro Hospital, General Surgery, selected the articles based on the titles and abstracts. A consultant undertook a thorough review of the articles considering the inclusion criteria and verified the selection.

All retrospective articles in English that analysed the complications of patients treated with laparoscopic subtotal cholecystectomy were included. In the articles in which some of the complications were not reported by the authors, these were considered not reported (NR) and therefore equal to zero.

The inclusion criteria for evaluating the selected articles were the total number of STCs both by open and laparoscopic surgery. The open procedure and the conversion from laparoscopy to open surgery were excluded from the present study.

Finally, all difficult laparoscopic cholecystectomies finalized by laparoscopic STC were considered.

The techniques used to complete difficult laparoscopic cholecystectomy have been recorded, considering the major techniques reported in the literature: Type A leaves part of the posterior wall attached to the liver, and the remaining gallbladder stump remains open; Type B is similar to A, but the stump is closed; Type C differs from methods A and B because it includes resection of both the anterior and posterior gallbladder walls. In method C, the pouch is closed, and drains are not used routinely compared to other methods; Type D is like Type C but with the stump open [14].

Complications were analytically recorded as early (by 30 days) and as late (more than 30 days). Complications were also analysed using the Dindo–Clavien classification [15]. Mortality at 30 days was studied.

## Results

**Fig. 1:**
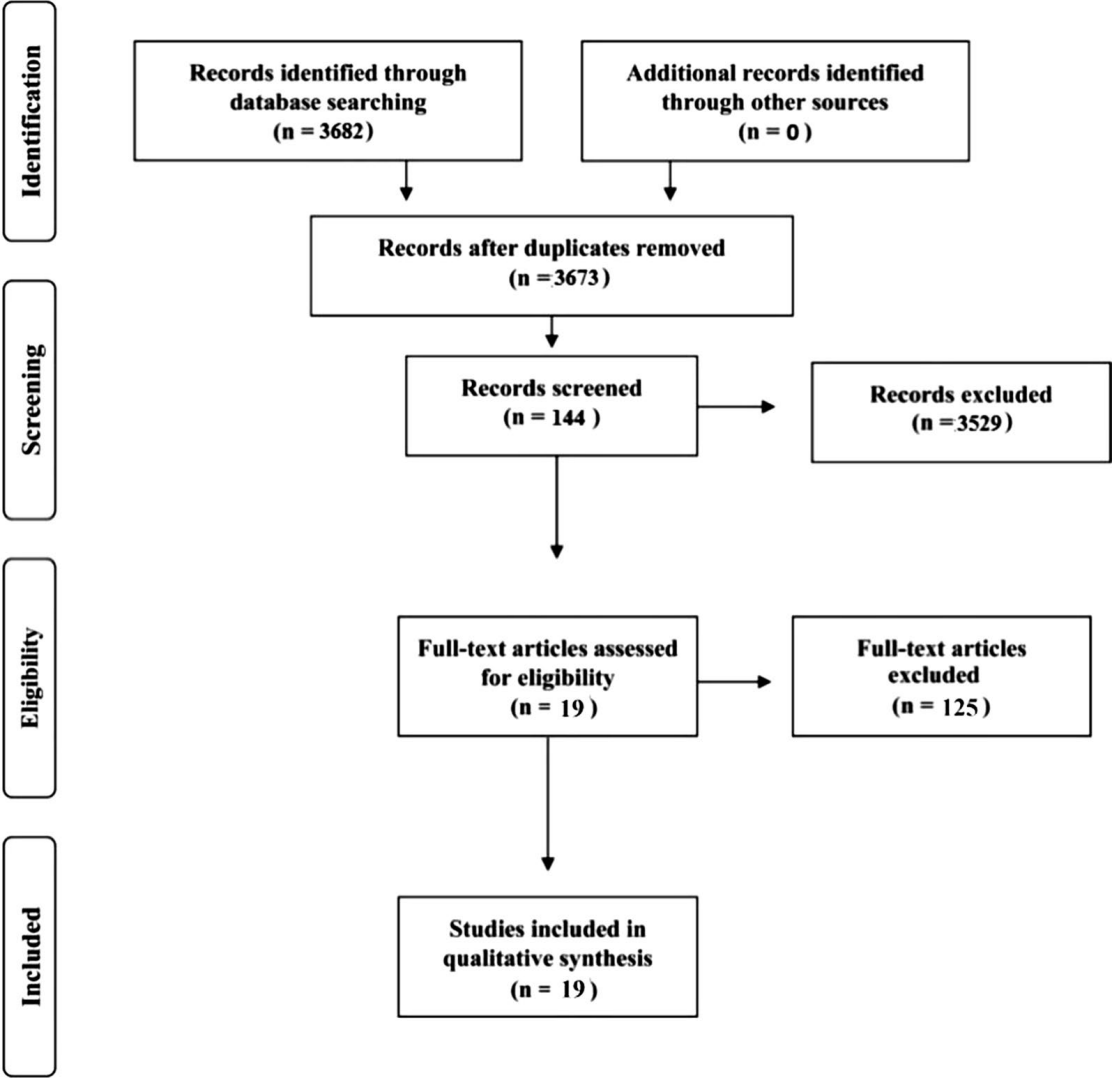
Algorithm used to screen the manuscripts

Using the terms applied, 3682 publications were identified. In the first screening by title and abstract, 3529 articles were excluded because the terms were used in a different context, and nine were excluded because they were repeated. One hundred and forty-four articles were reviewed. Of these, 118 were excluded because they did not meet the inclusion criteria, and seven because the authors did not clearly specify the type of technique used. Therefore, a total of 19 articles were included in the review [7–10, 14, 16–29] (Fig. 1).

The total number of patients undergoing cholecystectomy was 13,340 (100%). Of these, 4789 (35.9%) were not analysed by the authors of the related manuscript, and 121 patients (0.9%) underwent open cholecystectomy (OC). Both groups were excluded from the present study. A total of 8430 patients (63.2%) underwent laparoscopic cholecystectomy (LC). Of these last group of patients, 784 (5.9%) had a difficult laparoscopic cholecystectomy for cholecystitis; 106 patients (1.3%) of this last group were converted to open surgery and for this reason excluded from the present study.

Finally, 678 patients (8.0%) had a difficult cholecystectomy completed by subtotal cholecystectomy in laparoscopy, and they represent the core of the study (Table 1). The weighted average age was 59.3 years.

**Table 1:** Articles analysed in the literature to obtain the total number of STLC cases studied

References	Number of cholecystectomy	Not analysed	Open cholecystectomy	Laparoscopic cholecystectomy	Converted LAPARO-OPEN	Subtotal cholecystectomy
Michalowski [16]	340			340	24	29
Ransom [17]	125	125				8
Chowbey [18]	1680			1680	3	53
Beldi [9]	345			345	21	37
Sinha [19]	889	889				28
Horiuchi [20]	285			285		25
Philips [21]	1917			1917		26
Hubert [22]	552		52	500		39
Jeong [7]	1069	951	38	80		26
Kuwabara [23]	246			246		26
Kulen [8]	80			80	40	40
Harilingam [24]	993		14	979	13	64
Shin [25]	1107	21		1086		51
Abdallah [26]	373		2	371	3	65
Matsumura [27]	427		15	412	1	12
Ozcinar [10]	200	200				5
Abdelrahim [14]	109			109		17
Kohga [28]	290	290			1	42
Slater [29]	2313	2313				85
Total	13,340	4789	121	8430	106	678
Percentage (%)	100	35.9	0.9	63.2	1.3	8.0

The technique used to complete the LSTC was as follows: Type A in 8 articles (40.0%) for a total of 346 patients (51.0%); Type B in 7 articles (35.0%) for a total of 134 patients (19.8%); Type C in 5 articles (25.0%) for a total of 198 patients (29.2%); and Type D had no reported (0%) articles or patients.

The surgical complications are analytically reported in Table 2.

**Table 2:** Postoperative complications of STLC

References	No LSC	Type	Postoperative complications										
			Early compliance								Late complications		
			Bile leak	ERCP postoperative	Other	Subhepatic collections	Residual stone	Wound infection	Intra-abdominal infection	Haemorrhage	Port hernia	CBD Stenosis	Mortality at 30 days
Michalowski [16]	29	B	4		9	4		1					1
Ransom [17]	8	B											0
Chowbey [18]	53	C	3					4					
Beldi [9]	37	A	33	7	2	16	3	1	1	1	5		1
Sinha [19]	28	A	5	3	1				1				0
Horiuchi [20]	25	B	1						0	0			
Philips [21]	26	A	4	5	2		1		1		2		1
Hubert [22]	39	A		2	5		2	1					0
Jeong [7]	26	A	0				0	0	0	0			0
Kuwabara [23]	26	B	0	1			1	0	0	0			
Kulen [8]	40	A	0		1		0	0	0	0			0
Harilingam [24]	64	C	2	6			3					1	
Shin [25]	22	C	4					0	1				0
	29	B	6					0	1				0
Abdallah [26]	65	A	0	1	1			3	0				0
Matsumura [27]	12	B	1	2			1		0				0
Ozcinar [10]	5	B	0	1			1	0	0	0			0
Abdelrahim [14]	17	C	0	1			1	0	0		1		
Kohga [28]	42	C	4	3	2		3						0
Slater [29]	85	A	16	15	3	3	2						
Total	678		83	47	26	23	18	10	5	1	8	1	3
Percentage (%)	100		12.2	6.9	3.8	3.4	2.7	1.5	0.7	0.1	1.2	0.1	0.4

The major cause for early complications was bile leakage, which was found in 83 patients (12.2%). This is frequently reported in Type A with 58 cases (69.9%), followed by Type C with 13 cases (15.6%) and Type B with 12 cases (14.5%). These patients were treated with biliary stent placement by ERCP in 28 patients (33.7%) and in 34 patients (41.0%) with postoperative percutaneous abdominal drainage, invariably removed after approximately 7 days. In 21 patients (25.3%), biliary leakage resolved spontaneously, usually after a median of 7 days (range of 3–31 days).

Of the 678 patients analysed, 23 patients (3.4%) developed a subhepatic collection. Of these, 19 patients (82.6%) did not require any treatment and the remaining 4 patients (17.4%) underwent radiological drainage. Nineteen patients (82.6%) who developed a subhepatic collection underwent LSTC using technique Type A, 4 patients (17.4%) who developed a subhepatic collection after having the Type B technique, and none of the patients (0%) who developed a subhepatic collection were treated with technique Type C.

Intra-abdominal infections were found in 5 patients (0.7%). Three patients underwent reoperation, and all 3 patients were treated with Type A; one patient drained percutaneously; and one patient was treated with antibiotic treatment and did not need supplementary treatment. Wound infections (including port site infections) were reported in 10 patients (1.5%); they were treated locally by our clinic.

Residual stones in the CBD were found in 18 patients (2.7%), but only 13 cases documented postoperative endoscopic treatment in the 30 days after the procedure. One patient with an infected residual stone underwent a surgical procedure [9].

In total 47 patients were treated by ERCP; 27 patients (57.4%) that represent the majority of them were treated to position a stent for biliary leak; 13 were treated for CBD stones postoperatively; 5 patients were treated by ERCP but the authors did not specify the position of the stones evaluated; finally 1 patient was treated late for a biliary stenosis. Of the 13 patients treated by ERCP for CBD postoperatively stones, the authors did not specify if the stones were present preoperatively. Therefore, we cannot determine if there was migration of the stones during LSTC (Table 3).

**Table 3:** Major complications reported using STLC

References	Type	Bile leak				Subhepatic collections			ERCP postoperative			
		Number	Nothing	Drainage	Stent	Number	Nothing	Drainage	Number	Residual stone	Stent	Other
Michalowski [16]	B	4	3	1		4	2	2				
Ransom [17]	B											
Chowbey [18]	C	3		3								
Beldi [9]	A	33		29	4	16	15	1	7	3	4	
Sinha [19]	A	5	2		3				3		3	
Horiuchi [20]	B	1		1								
Philips [21]	A	4			4				5	1	4	
Hubert [22]	A								2	2		
Jeong [7]	A											
Kuwabara [23]	B								1	1		
Kulen [8]	A											
Harilingam [24]	C	2			2				6	3	2	1
Shin [25]	C	4	3		1							
	B	6	5		1							
Abdallah [26]	A								1			1
Matsumura [27]	B	1	1						2	1	1	
Ozcinar [10]	B								1	1		
Abdelrahim [14]	C								1	1		
Kohga [28]	C	4	4						3	3		
Slater [29]	A	16	3		13	3	2	1	15	2	13	
Total		83	21	34	28	23	19	4	47	18	27	2
Percentage (%)			25.3	41.0	33.7		82.6	17.4		38.3	57.4	4.3

Only one case of minor bile duct injury repaired intraoperatively (Bismuth Type I) was reported.

Other types of complications are described in Table 2.

Finally, 1 patient (0.1%) with common duct biliary stenosis [8] was reported as a late complication; port hernias were found in 8 patients (1.2%).

The Clavien–Dindo classification [15] revealed that there were four grade I, 27 grade II, 126 grade IIIa, 18 grade IIIb, no grade IV and 3 grade V outcomes (Table 4).

**Table 4:** Complications using Dindo–Clavien classification

Grades	Definition	PZ
Grade I	Any deviation from the normal postoperative course without the need for pharmacological treatment or surgical, endoscopic and radiological interventions. Allowed therapeutic regimens are as follows: drugs as antiemetics, antipyretics, analgetics, diuretics and electrolytes and physiotherapy. This grade also includes wound infections opened at the bedside	4
Grade II	Requiring pharmacological treatment with drugs other than such allowed for grade I complications. Blood transfusions and total parenteral nutrition are also included	27
Grade III	Requiring surgical, endoscopic or radiological intervention	
IIIa	Intervention not under general anaesthesia	126
IIIb	Intervention under general anaesthesia	18
Grade IV	Life-threatening complication (including CNS complications) * requiring IC/ICU-management	
IVa	Single organ dysfunction (including dialysis)	
IVb	Multiorgan dysfunction	
Grade V	Death of a patient	3

Mortality at 30 days was recorded in three patients (0.4%). Two patients died due to myocardial infarction after the procedure, and one patient died due to HIV-related complications. All three patients died during the hospitalization.

## Discussion

In recent years, there has been an increase in the LC rate, which went from 71.9% in 2003 to 86% in 2014. There was a corresponding increase in LSTC rates, which went from 0.12 to 0.28% [30]. As reported in the literature, in cases in which it is not possible to complete a laparoscopic subtotal cholecystectomy due to advanced fibrosis and inflammation, conversion to the open technique is preferable [31, 32]. All young surgeons mainly trained in laparoscopy have to consider it.

The incidence of difficult cholecystitis reported in the literature is 10–15% of the total cases of acute calculous cholecystitis [33]. This discrepancy depends on the method used to classify the difficulty of the surgical procedure. The major reasons to classify a cholecystectomy as difficult are the severity of the disease, the presence of adhesions with consequent anatomical alteration, the laparoscopic experience of the surgeon and the devices available for surgical treatment [34]. Severe inflammation of Calot's triangle can produce fibrosis with alteration of all anatomic landmarks and consequent risk of iatrogenic injury to the common hepatic duct, the common bile duct and the cystic duct [35–37]. According to the Tokyo 2018 guidelines, the degree of severity of acute cholecystitis correlates with an increased risk of bile duct injury (BDI) [35]. BDI leads to increased hospital costs and mortality rates and can require liver resection or even liver transplantation [35].

Various techniques have been reported in the literature to avoid BDI: obtaining a critical view of safety (CVS) [38], identifying Rouvière's sulcus [39], performing intraoperative cholangiogram (IOC) [40], performing intraoperative fluorescent cholangiogram using indocyanine green [41], and converting to an open procedure [7].

CVS is a method of identifying cystic structures (cystic artery and cystic duct) described by Strasberg. The term "CVS" was first coined in 1995 [42]. Three requirements are needed for CVS: 1) Calot's triangle must be cleared of fatty and fibrous tissue without exposing the common bile duct and the common hepatic duct, 2) the lower third of the gallbladder must be separated from the liver to expose plaque cysts, and 3) only two structures need to enter the gallbladder.

Rouvière's sulcus (RS), also called incisura hepatis dextra or Gans incisura, is a cleft in the liver that is located anterior to segment 1. The cystic duct and the cystic artery are located antero-superior to the sulcus, while the common bile duct is located under the sulcus [43].

Intraoperative cholangiography, by means of the transcystic infusion of contrast medium, allows the identification of the stones and the anatomy of the biliary system. Mirizzi described it for the first time in 1931 [44].

The intraoperative fluorescent cholangiogram using indocyanine green intravenously 30 min before the surgical procedure allows fluorescent images of the biliary system to be obtained [45].

A meta-analysis was recently published in which the use of indocyanine green fluorescent cholangiography (FC) during surgery considerably reduces bile duct lesions and conversion rates in open surgery compared to white light cholecystectomy alone [46], but no comparative studies are available. The cost of FC is less expensive in relation to IOC, on the opposite IOC is more available in country hospital in relation to FC [47].

All these methods permit the completion of a procedure without the risk of complications that can be detrimental to patients throughout their lives. However, when none of these techniques can be used for safety concerns, then Calot’s triangle should not be approached, and thus, subtotal cholecystectomy must be performed.

In 1950, the partial cholecystectomy technique was described in which three-quarters of the gallbladder was removed, leaving a portion of the posterior wall attached to the liver without electrocoagulating the mucosa. The cystic duct was not closed [48]. In 1985, the subtotal cholecystectomy technique was modified: the posterior wall of the gallbladder was left attached to the liver, and the cystic duct was closed with a purse-string technique [49].

Currently, the most reputed method to solve this problem is subtotal cholecystectomy removing both the anterior and posterior walls with suturing of the infundibulum. This method is reported for open, open converted or laparoscopic procedures [6, 8, 50]. A different method called partial cholecystectomy consists of resection of the fundus [48, 51], but it has been abandoned for the complications reported [52].

Articles with a limited number of patients report that subtotal cholecystectomy is associated with a reduction in bile duct injuries and conversion rate but report an increase in bile leaks and retained stones that require reintervention [9, 52]. ERCP can be applied not only in cases of biliary leakage but also for clearance of the biliary tract from residual stones, which can increase the common bile duct pressure and favour leakage from the cystic duct, especially if left open. These two complications were solved using ERCP in 95% of the population [53, 54]. Early or late, this procedure can be applied for stenosis of the biliary tract post cholecystectomy.

Biliary leakage represents the most frequent complication of incomplete resection of the gallbladder wall in cases of difficult acute cholecystectomy treated with subtotal cholecystectomy. This complication is rarely fatal but requires correct treatment. If bile leakage does not stop spontaneously seven days postoperatively, the possible treatments are endoscopic biliary sphincterotomy [55], endoscopic plastic stent [54], and a fully covered self-expanding metal stent [54].

The method that has the majority of these complications is subtotal cholecystectomy Type A, probably because the posterior wall remains attached to the liver and the remnant anterior wall is left open. Additionally, Types B and C have a possibility of this complication but with lower percentage. This is likely due to staplers being used on walls that are thickened due to inflammation. To try to reduce postoperative fistulas after fenestrating, the omental plugging technique (OPT) was developed in 2011. This technique consists of placing a piece of omentum on the stump of the gallbladder to prevent the leakage of bile, but in effect, the results are ineffective [6, 56]. In our study, it was well clarified that leaving the wall of the gallbladder open was the major risk for biliary leak complications. As a result, this technique should be the last resort for treating these patients. Of course, ERCP or the positioning of a stent may treat all kinds of biliary leaks, but this represents an increased cost and a poorer quality of life for patients who need to visit outpatient clinics for a period of time.

Subhepatic collections are usually described as a non-infective fluid collection, but an abscess can also be present. Some of these collections can be resorbed without any clinical signs or complications. In our study, it was well demonstrated that these complications are strictly related to the gallbladder wall being left open. When this procedure is applied, it becomes mandatory to drain the abdomen at the end of the surgical procedure. First to avoid more complications and second to understand the patient's needs and timing for treatment. Abdominal drainage after difficult laparoscopic cholecystectomy prevents abdominal fluid collection, its infectious process and the consequent treatment with increase in hospitalization, costs and deterioration of patient quality of life [22].

A small number of patients who underwent drainage developed a subhepatic collection because drainage was likely removed early. When a subhepatic collection is formed, a radiological intervention is needed, and in most difficult cases, reoperation may also be necessary.

Intra-abdominal infection in cases of difficult cholecystectomies depends on the preoperative situation and the intraoperative status. Intraoperatively, it is of the utmost importance to wash and clean at the end of the procedure and to position appropriate drainage to mitigate any collections and consequent infection. In patients with intra-abdominal infection or subhepatic collections, the quality of life can also be modified.

Haemorrhage can occur when the wall is inflamed. In this case, the posterior wall probably has to be left in place when applying technique B, which is safe both for leakage and bleeding.

Wound infection is strictly related to free bile in the abdomen due to the difficulty of the procedure. This can occur due to contact between trocar extraction and wound infection. This complication is not related to the different methods of subtotal cholecystectomy.

Residual stones are usually found postoperatively in the common bile duct. These stones can migrate during the procedure or be concomitant with inflammation of the gallbladder. In the present study, the number of patients who preoperatively suffered from common bile duct stones was not reported. Usually, ERCP resolves the problem definitively. Residual stones can also remain in the stump when both the anterior and posterior walls are sutured. It is of utmost importance during the procedure to explore the remnant cavity before suturing. If the cystic duct can be cannulated, intraoperative cholangiography can be performed, and if small stones are identified in the cystic duct, tentative elimination of these small stones with low-pressure irrigation of the cystic duct must be performed [14]. Furthermore, a large residual stump of the gallbladder can recreate the lumen, and therefore, new stones can form.

Bile duct injuries can be a significant complication in this type of surgery. Prevention of the lesions with conversion from laparoscopic to open, or the opinion of older surgeon in case of difficulties is strongly recommended. BDI recognized and repaired intraoperatively can improve immediate and late results [57].

Mortality is a very rare complication. In our research, only a few cases have been reported but for a cause unrelated to the procedure.

The limitations of our study are given by the heterogeneity of the techniques used for LSC and the lack of a long-term follow-up analysing the related complications.

## Conclusion

When performing closure of the gallbladder stump, suturing the anterior residual of both anterior and posterior wall represents the best method to have fewer complications. Complications, if not lethal, decrease the patient’s quality of life. Intraoperatively, it is of utmost importance to carefully expose the gallbladder stump to avoid left-in-place stones, wash the entire cavity and drain the abdomen.

## Data Availability

The datasets used and/or analysed during the current study are available from the corresponding author upon reasonable request.

## Abbreviations

LSTC: Laparoscopic subtotal cholecystectomy
STC: Subtotal cholecystectomy
OC: Open cholecystectomy
LC: Laparoscopic cholecystectomy
ERCP: Endoscopic retrograde cholangiopancreatography
CBD: Common bile duct
CVS: Critical view of safety
RS: Rouvière's sulcus
OPT: Omental plugging technique
